# Electroacupuncture for post-thoracotomy pain: Protocol for a systematic review and meta-analysis

**DOI:** 10.1097/MD.0000000000019312

**Published:** 2020-02-28

**Authors:** Sohyeon Park, Ji-Hyang Gu, Hye-Kyoung Jang, Min-Seok Oh, Eun-Jung Lee, In Chul Jung

**Affiliations:** a College of Korean Medicine; b Department of Korean Rehabilitation Medicine, College of Korean Medicine, Daejeon University, Dong-Gu; c Clinical Trial Center, Dunsan Korean Medicine Hospital of Daejeon University, Seo-Gu Daejeon; d Department of Oriental Neuropsychiatry, College of Korean Medicine, Daejeon University, Dong-Gu, Daejeon, Republic of Korea.

**Keywords:** electroacupuncture, postoperative pain management, thoracotomy

## Abstract

**Background:**

Thoracotomy is a common surgical procedure used in cases such as trauma and cancer resection. It is an invasive procedure in which incisions are made in the chest wall to gain access to the chest. Therefore, it often produces intense postoperative pain. Electroacupuncture has been known for its analgesic effects in various conditions, including cases of postoperative pain. This protocol design is for a systematic review and meta-analysis to gather evidence and investigate the analgesic effects of electroacupuncture in pain after thoracotomy.

**Methods:**

The studies for the systematic review will be searched with keywords on the following 10 databases: PubMed, Cochrane Library (CENTRAL), EMBASE, MEDLINE, Google Scholar, CNKI, KoreaMed, KMBASE, KISS, and OASIS. The search will be done without language restrictions. Only the randomized controlled trials that meet the eligibility criteria will be finally included in the study. The quality of the study will be assessed using the Cochrane Collaborations’ risk-of-bias tool, and Cochrane's software RevMan 5.3 will be used for meta-analysis.

**Results:**

The designed study will provide a systematic review and meta-analysis of the searched and randomized controlled trials that meet the eligibility criteria. Meta-analysis will be performed with pain scores as the main outcome measure, and they may also be performed with additional outcomes. The qualitative and quantitative data synthesis is expected to provide high quality evidence to judge the pain management effect of electroacupuncture for patients who underwent thoracotomy.

**Conclusion:**

The conclusion of this systematic review and meta-analysis will provide evidence to judge whether electroacupuncture is an effective analgesic treatment option for patients suffering with post-thoracotomy pain.

**PROSPERO registration number:**

CRD42019142157.

## 1 Introduction

Thoracotomy is an invasive surgical procedure to gain access into the pleural space, and it is performed in cases such as cancer resections and trauma. Patients who undergo thoracotomy often suffer from severe postoperative pain due to its invasive nature.^[1]^ Post-thoracotomy pain management is also important because lack of proper management may lead to prolonged chronic pain.^[2,3]^

There are several ways to manage postoperative pain, and analgesics including opioids are a major part of the process. Opioids are medications that provide a strong analgesic effect by interacting with mu opioid receptors.^[4]^ However, there are several reported side effects of the medication, including respiratory depression, cough suppression, reduced intestinal motility, nausea, vomiting, and urinary retention.^[5]^ Also, opioids are highly addictive. According to the National Institute of Drug Abuse, drug overdose deaths in the United States involving prescription opioids rose from 3442 in 1999 to 17,029 in 2017.^[6]^ Therefore, several other treatment options have been sought and developed to help reduce the amount of opioids used.

Electroacupuncture (EA) has been known for its analgesic effects, and there are several studies proving the pain management effects of EA in different occasions, including postoperative conditions.^[7]^ The objective of the systematic review is to provide evidence to evaluate the analgesic effect of EA on postoperative pain compared to conventional analgesia.

A detailed study protocol with an appropriate review question and eligibility criteria for systematic review can help researchers perform a thorough study selection, which is critical to the final quality of the evidence.^[8–10]^ The aim of the protocol is to set a proper guideline for the systematic review and meta-analysis on the effects of EA on post-thoracotomy pain.

## 2 Methods

This study protocol is registered in the International Prospective Register of Systematic Reviews (PROSPERO) as CRD42019142157.

### 2.1 Eligibility criteria for study inclusion

#### 2.1.1 Types of studies

Randomized controlled trials (RCT) will be included in the research. Other types of studies such as case reports, case series, literature review, and uncontrolled trials will not be included. There will be no language restrictions during the search, but all search words will be in English.

#### 2.1.2 Types of participants

Patients who received thoracotomy will be included. There will be no restrictions on age, gender, ethnicity, type of surgery or patient's disease for the selection process. The following terms will be searched in combination to include the thoracotomy cases: thoracotomy, thoracic surgery, lobectomy, pneumonectomy, esophagectomy, open-heart surgery, and cardiac surgery.

#### 2.1.3 Types of interventions

The treatment group will receive EA without any limits on the type of needles, frequency, or applied area. Other types of acupuncture without electrical stimulation will not be included.

#### 2.1.4 Types of controls

The control group will include all treatments except for EA. It may include sham EA, no treatment group, and other conventional analgesia including general anesthesia and analgesics.

#### 2.1.5 Types of outcome measure

The primary outcome will yield numerical pain measuring scores including visual analog scale (VAS). Additional outcome measures such as total amount of postoperative analgesics, the amount of released chemicals that have analgesic effects (endogenous analgesics, serotonin, etc), and other indicators regarding pain management may be considered as secondary or tertiary outcome measures for meta-analysis if the data are sufficient for synthesis.

### 2.2 Search methods for the identification of studies

“Is EA combined analgesia effective in reducing post-thoracotomy pain compared to analgesia without EA?” This will be used as the review question and fits the PICO form. The following 10 databases will be searched: PubMed, Cochrane Library (CENTRAL), EMBASE, MEDLINE, Google Scholar, CNKI, KoreaMed, KMBASE, KISS, and OASIS. Considering the characteristics of the intervention, 1 Chinese database (CNKI) and 4 Korean databases (Koreamed, KMBASE, KISS, and OASIS) will be included in the list of databases. Keywords will be searched in a combined form of 3 major categories: thoracotomy, postoperative pain, and acupuncture. The combination of search terms in each category will be as follows: (“Thoracotomy” OR “thoracic surgery” OR “lobectomy” OR “pneumonectomy” OR “esophagectomy” OR “open heart surgery” OR “cardiac surgery”) AND (“pain” OR “postoperative” OR “perioperative” OR “anagesia” OR “analges∗”) AND (“acupuncture” OR “acupressure” OR “acupoint” OR “acup∗” OR “electroacupuncture”). Trials including patients who received video-assisted thoracic surgeries will be excluded using the phrase (NOT “Video-assisted”). The search will aim for RCTs, which includes key terms in titles or abstracts. The search format will be adjusted based on the system of each database (Table 1).

**Table 1:**
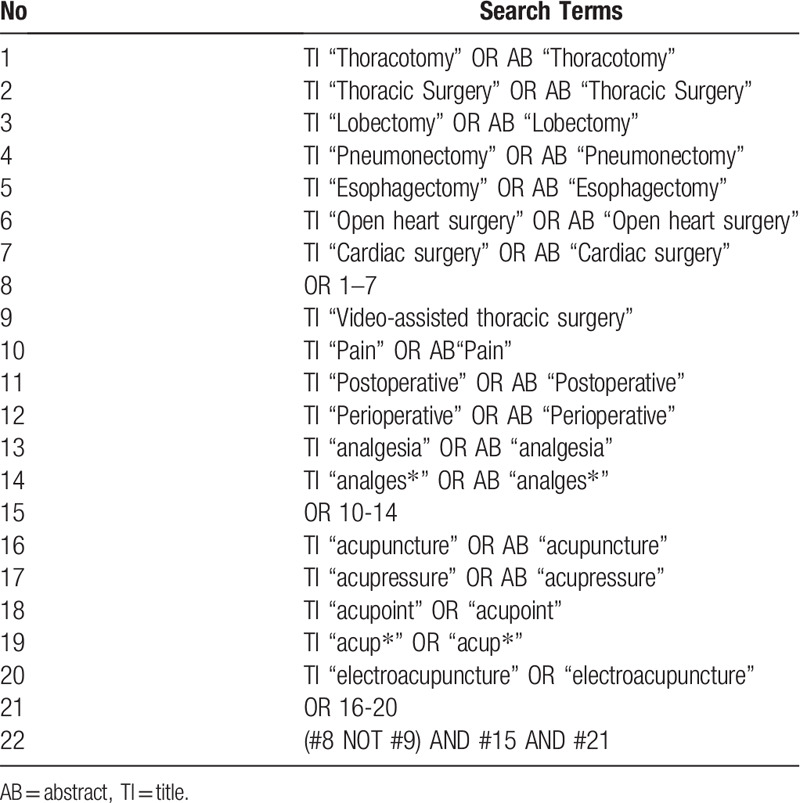
Search strategy for MEDLINE.

### 2.3 Data collection and analysis

#### 2.3.1 Selection of studies

**Figure 1:**
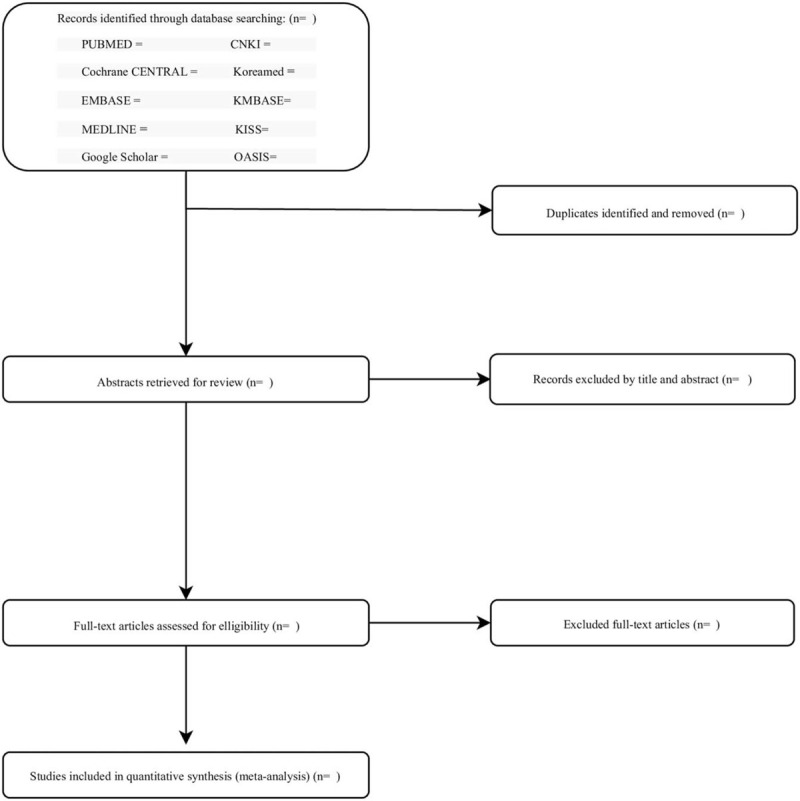
Flow chart of study selection process.

Two researchers will independently perform the following process. After gathering the search results from the 10 databases, duplicates will be identified and removed using the software EndNote. Then, studies that do not meet the eligibility criteria judging by their titles and abstracts will be excluded. Full texts of the remaining studies will be retrieved and assessed for the final selection process. Any disagreements during the inclusion process will be addressed in a group discussion including a third researcher (Fig. 1).

#### 2.3.2 Data extraction and management

Two researchers will independently extract data. The extracted data will include author, year of publication, study design, type of surgery, sample size (of each group), baseline characteristic differences between the treatment and control groups, intervention of the treatment and control groups, outcomes, results, and conclusions. Any discrepancies and doubts will be clarified with the help of a third researcher. The extracted data will be presented in a summary table.

#### 2.3.3 Assessment of risk of bias in the included studies

The Cochrane risk-of-bias tool in the Cochrane Collaboration's RevMan 5.3 software will be used in the quality assessment process. The tool consists of the following 7 domains: random sequence bias (selection bias), allocation concealment (selection bias), blinding of participants and personnel (performance bias), blinding of outcome assessment (detection bias), incomplete outcome data (attrition bias), selective reporting (reporting bias), and other bias. Two researchers will independently judge the quality in each domain of the tool for each study, and group discussions will be held to resolve any discrepancies in consultation with a third researcher. The quality assessment results will be illustrated in a risk-of-bias graph and a risk-of-bias summary table.

#### 2.3.4 Measures of treatment effect

For continuous data, the mean difference will be calculated with 95% confidence interval (CI). If the outcomes are measured with different scales, the standard mean difference will be calculated with 95% CI. For dichotomous data, the odds ratio (OR) or risk ratio will be used with 95% CI.

#### 2.3.5 Dealing with missing data

If the full text of an article is unavailable, a researcher will try to contact the author for retrieval. If an attempt to contact the author fails, the unavailable data will be excluded from the analysis.

#### 2.3.6 Assessment for heterogeneity

Higgins *I*^2^ test will be performed to estimate statistical heterogeneity. If *I*^2^ ≥ 50%, the heterogeneity will generally be considered high. If heterogeneity turns out to be high, a random-effects model should be chosen, and subgroup analysis will be considered.

#### 2.3.7 Assessment of reporting bias

If more than 10 RCTs are included in a meta-analysis, a funnel plot will be presented to assess the reporting bias.

#### 2.3.8 Data synthesis

The Cochrane Collaboration's software RevMan 5.3 will be used in the meta-analysis. The primary outcome measure for the data synthesis will be numerical pain scores such as VAS, representing the patients’ pain after surgery. Additional outcome measures, such as total dose of analgesics or released chemicals related to analgesia, may be used for additional meta-analyses if multiple RCTs provide statistically significant amounts of data. If study results come from different population sizes, a random-effects model will be chosen. Otherwise, a fixed-effects model can be chosen. The synthesized result will be visually represented in a forest plot. If quantitative synthesis is not appropriate, only a descriptive analysis may be done.

#### 2.3.9 Subgroup analysis

If the included studies show high heterogeneity and the number of included trials is sufficient, subgroups will be made based on the type of control group.

#### 2.3.10 Sensitivity analysis

Sensitivity analysis will be carried out to evaluate the robustness of the meta-analysis by repeating the meta-analysis when it includes a vague or arbitrary decision-making process.

#### 2.3.11 Grading the quality of evidence

Grading of recommendations assessment, development and evaluation will be used to evaluate the confidence in cumulative evidence. The strength of each evidence will be rated “Very low,” “Low,” “Moderate” or “High.”

## 3 Dissemination and ethics

This study is not required to receive ethical approval since the data used in the research are not individualized. The results will be disseminated in a peer-reviewed journal and may be presented in related conferences.

## 4 Discussion

Thoracotomy is an invasive surgical procedure which causes intense and often prolonged postoperative pain.^[1]^ The objective of this systematic review and meta-analysis is to provide high quality evidence to judge whether EA is an effective form of analgesia on post-thoracotomy patients compared to conventional analgesics.

To perform a high-quality systematic review and meta-analysis, a rigorously designed protocol is needed. The aim of this protocol is to provide a proper guideline for the systematic review and meta-analysis by proposing detailed eligibility criteria and appropriate data synthesizing methods.

There may be some limitations in this study protocol. First, only 1 Chinese database and 4 Korean databases were included in the list of databases and they will all be searched with English terms. As a result, studies that do not provide English titles or abstracts will not be included, and some Japanese RCTs may be missed. Second, the quality of the research will be affected by the amount and the quality of the search result. If there is a high number of rigorously designed previous studies that meet the eligibility criteria, the systematic review and meta-analysis will be able to provide high-quality evidence. Otherwise, it may not be possible to provide reliable evidence because biases in primary studies are often reflected in the meta-analysis.^[11,12]^ Still, it could make researchers aware that several rigorously designed RCTs regarding the effect of EA on post-thoracotomy pain are needed. Ideally, several high-quality RCTs will be found and included in the study so that a high-quality data synthesis can be performed, thus helping clinicians judge whether EA is an effective option for inclusion in the post-thoracotomy pain management plan.

## Author contributions

**Conceptualization:** Eun-Jung Lee, In Chul Jung.

**Methodology:** Sohyeon Park, Ji-Hyang Gu.

**Supervision:** Min-Seok Oh, Eun-Jung Lee, In Chul Jung.

**Writing – original draft:** Sohyeon Park, Ji-Hyang Gu.

**Writing – review and editing:** Sohyeon Park, Hye-Kyoung Jang.
